# Stress Induced Hyperglycemia in the Context of Acute Coronary Syndrome: Definitions, Interventions, and Underlying Mechanisms

**DOI:** 10.3389/fcvm.2021.676892

**Published:** 2021-05-12

**Authors:** Mingmin Li, Guo Chen, Yingqing Feng, Xuyu He

**Affiliations:** Department of Cardiology, Guangdong Provincial Key Laboratory of Coronary Heart Disease Prevention, Guangdong Cardiovascular Institute, Guangdong Provincial People's Hospital, Guangdong Academy of Medical Sciences, Guangzhou, China

**Keywords:** stress induced hyperglycemia, acute coronary syndrome, admission blood glucose, intensive glucose control, oxidative stress

## Abstract

Elevation of glucose level in response to acute coronary syndrome (ACS) has been recognized as stress induced hyperglycemia (SIH). Plenty of clinical studies have documented that SIH occurs very common in patients hospitalized with ACS, even in those without previously known diabetes mellitus. The association between elevated blood glucose levels with adverse outcome in the ACS setting is well-established. Yet, the precise definition of SIH in the context of ACS remains controversial, bringing confusions about clinical management strategy. Several randomized trials aimed to evaluate the effect of insulin-based therapy on outcomes of ACS patients failed to demonstrate a consistent benefit of intensive glucose control. Mechanisms underlying detrimental effects of SIH on patients with ACS are undetermined, oxidative stress might play an important role in the upstream pathways leading to subsequent harmful effects on cardiovascular system. This review aims to discuss various definitions of SIH and their values in predicting adverse outcome in the context of ACS, as well as the effect of intensive glucose control on clinical outcome. Finally, a glimpse of the underlying mechanisms is briefly discussed.

## Introduction

Stress-induced hyperglycemia (SIH) is an acute response of the bodies to many critical illnesses, including acute coronary syndromes (ACS) (1). Many observational studies have documented that hyperglycemia occurs frequently among patients hospitalized with ACS, even those without diabetes mellitus (1–3). It has been well-established that elevated glucose is associated with increased in-hospital and long-term mortality in ACS patients, especially in non-diabetic patients (4–10). Yet, hyperglycemia is not identified as an independent risk factor of ACS to date. Many questions regarding the relationship between SIH and ACS remain unclarified. For example, which glucose metrics, such as the blood glucose on admission (ABG), the average blood glucose, or the glucose variability (GV), is the most appropriate measurement of hyperglycemia and best correlated to the poor outcome; what the cut-off values that precisely define SIH are; whether there's any difference between cut-off values in the presence and absence of recognized diabetes mellitus; whether glucose-lowering therapy can improve the prognosis of ACS patients with SIH; and finally, what the mechanisms underlying SIH in the ACS setting are. This article aims to address some of the undetermined issues based on the present available data.

## Defining SIH in the Context of ACS

### Defining SIH With ABG

Despite numerous studies regarding SIH have been published, there's currently no uniform definition for SIH in the setting of ACS. Most early studies defined hyperglycemia by the first available glucose value or ABG (1, 4, 5, 8, 11–13). The most acceptable description of ABG refers to the first acquired blood glucose within 24 h of admission (1, 7). Nevertheless, the cut-point of ABG used to define hyperglycemia in patients with ACS was different from study to study. Back to 2008, the American Heart Association (AHA) Scientific Statement on Hyperglycemia and Acute Coronary Syndrome suggested using an ABG level >140 mg/dL as the definition of hyperglycemia under such circumstances (6). Evidence behind this recommendation mainly came from retrospective observational studies. In a national retrospective study of 141680 elderly acute myocardial infarction (AMI) patients, non-diabetic patients with higher ABG (range from>110 to 140 mg/dL) had increased risk of both 30-day and 1-year mortality compared with patients whose ABG was ≤ 110 mg/dL. In contrast, increased mortality was observed only in those with an ABG >240 mg/dL among diabetic AMI patients (4). Another study found AMI patients with a baseline glucose level <140 mg/dL had lower 30-day mortality compared to those with higher baseline glucose (14). Similarly, Buturlin et al. reported in a recent study of 4,520 ACS patients that mildly elevated ABG with glucose levels <140 mg/dL was not independently associated with increased 1-year mortality in non-diabetic patients (15). In a meta-analysis of 15 relatively small studies which discussed the association between ABG and ACS outcomes, Capes et al. indicated that among non-diabetic patients with AMI, the relative risk of in-hospital death in those with an ABG level >110 mg/dL was 3.9 compared with that of patients who were normoglycemic. On the contrary, among diabetic AMI patients, a greater risk of in-hospital death was observed only in patients whose ABG level ≥180 mg/dL (1). The phenomenon that cut-off values of ABG for predicting adverse outcome differed between diabetic and non-diabetic AMI patients was reported in similar studies (4, 8). An appropriate reason for such discrepancy might be the unawareness of different baseline glucose metabolic status between diabetic and non-diabetic patients. The ABG level is influenced by both acute physiological stress and chronic baseline glycemic levels, especially in patients with established diabetes mellitus. It's obviously that using a single cut-off value of ABG to define SIH, regardless of the previous glucose metabolic status, is not compelling. Therefore, new metrics including glycemic gap and stress hyperglycemia ratio (SHR), which eliminate the interference of chronic glycemic levels, were introduced (16–18).

### Defining SIH With Glycemic GAP/SHR

The glycemic gap is calculated from the ABG minus the HbA1c derived average glucose level. A recent study showed that glycemic gap instead of ABG was associated with increased mortality (9). Similarly, SHR is calculated from ABG divided by the HbA1c derived average glucose level, which is also expressed as acute-to-chronic glycemic ratio in some articles. In a prospective study including 1,553 AMI patients, the prognostic power of glycemic ratio for in-hospital mortality was particularly evident in diabetic patients. However, among non-diabetic patients, both glycemic ratio and ABG had a similar prognostic accuracy (17). Another study of patients with ST-segment elevation myocardial infarction (STEMI) found that the glycemic ratio was closely associated with an increased risk for poor in-hospital outcome among both diabetic and non-diabetic patients (19). In contrast, ABG showed an association with poor in-hospital outcome only in non-diabetic patients. A recent randomized study evaluated the predictive value of SHR for long-term outcome in both diabetic and non-diabetic patients with STEMI. It included 6,287 STEMI patients and followed up over 5 years, and finally demonstrated that high SHR was significantly associated with worse long-term outcome in non-diabetic, instead of diabetic patients (10). Coincidently, Yang et al. reported in an AMI cohort that patients with a high SHR were at increased risk for long-term MACCE, defined as composites of all-cause death, non-fatal myocardial infarction, and non-fatal stroke (20). Again, when the same analysis was applied to diabetic patients, the risk of MACCE did not differ between patients with and without a high SHR. Hence, the predictive value of SHR was similar among diabetic and non-diabetic patients for in-hospital outcomes, but differed for long-term outcomes. The underlying mechanisms is unknown, one possible explanation might be the effect of SIH is masked by diabetes itself, given the fact that diabetes contributes to poor long-term prognosis in AMI patients (10).

### Defining SIH With GV

Both ABG and SHR are derived from one blood glucose test. The nature of the metrics determines that it cannot reflect the full profile of glucose swings in the ACS setting. Patients with similar mean glucose levels can have markedly different glucose excursions. Meanwhile, glucose fluctuations can exert deleterious effects on both endothelial function and oxidative stress (21, 22). Previous studies reported increasing GV conferred a higher risk of mortality among critically ill patients, independent from mean glucose levels (23). Gong Su et al. demonstrated in an AMI cohort that GV, indicated as the mean amplitude of glycemic excursions (MAGE), was associated with increased risk of MACE instead of ABG or HbA1c (24). In a further study of 256 non-diabetic STEMI patients, high GV but not ABG was turned out to be associated with 3-month MACE (25). Subsequent studies emerged with similar conclusions that GV was a predictor of prognosis in patients with ACS regardless of the diabetic status (26–28). In addition, an elevated GV was suggested to be associated with hypoglycemia, an independent risk factor for patients with coronary artery disease (29, 30). Yet, among several methods to quantify GV, such as standard deviation, MAGE and coefficient of variation, there is no universally accepted “gold standard.” Given different methods being utilized in studies, the results should be interpreted with caution. Besides, there were a few studies focusing on other metrics, such as fasting glucose (FG). Considering the definition of FG used in these studies, it seems to be an alternative index for ABG. However, FG within 24 h of admission was reported to be associated with both increased short and long-term mortalities only in diabetic patients with ACS (31–33).

With present methodology, it seems unable to describe the complete profile of SIH in the ACS setting by utilizing a single glucose metrics. Moreover, an optimal definition of SIH should have a similar accuracy in predicting the cardiovascular outcomes among both diabetic and non-diabetic patients. Further investigations regarding how to precisely define or draw the outline of SIH are in demand.

## Interventions

Although it's widely accepted that ACS patients presenting with hyperglycemia are at increased risk for adverse outcome, it remains to be illustrated whether hyperglycemia is a direct mediator of poor outcome, or it's simply a marker indicating a greater disease severity. To address the issue, large randomized clinical trials of glucose control in hospitalized ACS patients are requisite. In contrast to plenty of clinical trials of target-driven glucose control in chronic hyperglycemia patients, a few trials exploring the optimal glycemic target for ACS patients have been performed (Table 1).

**Table 1:** Randomized trials designed to compare effect of intensive glycemic control with that of standard therapy in patients presenting with ACS and associated SIH.

**Clinical trial (year)**	**Study population**	**Number of patients** **(percentage of patients without known diabetes)**	**Admission glycemia (mg/dL)**	**Intervention glycemic target** ** (mg/dL)**	**Achieved glycemic target (intervention vs. control)** ** (mg/dL)**	**Primary endpoint**	**Result**
DIGAMI (1995)	AMI	620 (13%)	>198 mg/dL	126–180 mg/dL in acute phase	148 vs. 162 mg/dL at discharge^*^	Mortality at 3 months	NS
DIGAMI 2 (2005)	ACS	1,253 (NA)	>198 mg/dL	Group 1: 90–126 mg/dL(fasting), <180 mg/dL (non-fasting) Group 2: 126–180 mg/dL	163.8 vs. 163.8 mg/dL during first 24 h (NS)	All-cause mortality difference between group 1 and 2	NS
					A1c ~6.8 vs. 6.8% by the end of 2-year follow-up (NS)		
HI-5 (2006)	AMI	240 (52%)	≥140 mg/dL	<140 mg/dL	149.4 vs. 162 mg/dL during first 24 h (NS)	Mortality at in-hospital stage, 3 and 6 months	NS
					A1C 6.9 vs. 6.8% at 3 months (NS)		
					A1C 7.4 vs. 7.0 at 6 months (NS)		
Marfella (2009)	AMI (CABG)	50 (58%)	≥140 mg/dL	80–140 mg/dL for intervention arm 180–200 mg/dL for control arm	162.7 vs. 192.4 mg/dL^*^	LVEF, oxidative stress, apoptosis	↑LVEF^*^ ↓oxidative stress and apoptosis^*^
Marfella (2012)	AMI (CABG)	50 (62%)	>140 mg/dL	80–140 mg/dL for intervention arm	160.9 vs. 193.9 mg/dL^*^	Myocardial regeneration	↑Myocardial regeneration^*^
				180–200 mg/dL for control arm			
Marfella (2012)	STEMI (pPCI)	165 (53%)	≥140 mg/dL	80–140 mg/dL for intervention arm	145 vs. 191 mg/dL^*^	ISR	↓ISR
				180–200 mg/dL for control arm			
Marfella (2013)	STEMI (pPCI)	106 (62%)	≥140 mg/dL	80–140 mg/dL for intervention arm	144 vs. 201 mg/dL^**^	Myocardial salvage	↑Myocardial salvage
				180–200 mg/dL for control arm			
RECREATE (2012)	STEMI	287 (72%)	≥144 mg/dL	90–117 mg/dL	117.5 vs. 142.9 mg/dL^**^	Difference in mean glucose levels at 24 h	↓Glycemia
BIOMArKS2 (2013)	ACS (pPCI)	280 (90%)	140–288 mg/dL	85–110 mg/dL	112 vs. ≈130 mg/dL^**^	hsTropT 72 h after admission	NS

*AMI, acute myocardial infarction; ACS, acute coronary syndrome; STEMI, ST-elevation myocardial infarction; NA, not available; NS, not significant*.

* *p < 0.05;*

** *p < 0.001; CABG, coronary artery bypass sugery; pPCI, primary percutaneous coronary intervention; LVEF, left ventricular ejection fraction; ISR, in-stent restenosis; hsTropT, high-sensitive troponin T-value*.

To our knowledge, DIGAMI was the first randomized clinical trial designed to evaluate the effect of intensive glucose control in AMI patients presenting with SIH. A total of 620 patients presenting with AMI, either had recognized diabetes mellitus or had a blood glucose level >11 mmol/L without diabetes, were enrolled (34). Patients were randomized into intervention arm with insulin-glucose infusion followed by multidose subcutaneous insulin and control arm with conventional therapy. The primary endpoint was all-cause mortality at 3 months. Patients from the insulin arm had significantly lower glucose levels compared to the control arm during the interventional period. Although there was no difference between two treatment groups for the primary outcome, reduced all-cause mortality was observed in the insulin arm at both 1- and 3.4-year follow up points (35). Nevertheless, given that over 80% of the patients had recognized diabetes mellitus and the insulin treatment lasted 3 months, it's hard to tell whether acute or chronic intensive glucose control contributed more to the reduced mortality. Although similar studies emerged subsequently, DIGAMI was the only trial demonstrating a survival benefit from intensive glucose control. The following study DIGAMI 2 was performed to compare the effects of 3 different treatment strategies in diabetic patients with AMI. Unexpectedly, no difference in the glucose control was achieved between the treatment groups, and it failed to demonstrate early and continued insulin-based intense glucose control could reduce mortality (36).

In the HI-5 study, 40% of the enrolled AMI patients were hyperglycemic without known diabetes. Patients were randomized to receive either insulin-based therapy or conventional therapy (37). There was no difference between two treatment arms in the mean 24-h blood glucose level. Despite a lower incidence of cardiac failure and reinfarction in the intervention arm within 3 months, HI-5 failed to demonstrate a reduced mortality at the in-hospital stage, 3 or 6 months. Nerenberg et al. enrolled 287 patients with AMI and hyperglycemia and randomly assigned them to either tight glucose control or usual care (38). At 24 h, patients from the tight glucose control arm had significant lower glucose levels compared to those from control arm, yet the 90-day mortality didn't differ between two arms. Besides, in a study by Marfella et al., 50 hyperglycemic patients diagnosed with AMI were randomized to intensive glycemic control (target glucose level 80–140 mg/dL) or conventional glycemic control for almost 3 days before surgery (39). Compared to the control group, patients in the intensive group had higher ejection fraction, less oxidative stress, less inflammation in peri-infarcted specimens. In their following studies, tight glucose control in hyperglycemic patients with STEMI brought benefits to both myocardial salvage and in-stent restenosis at 6 months after onset (40, 41).

Given the inconsistent results of clinical trials about glucose control in AMI patients, de Mulder et al. realized the inappropriate glucose target might be the problem. In their randomized trial BIOMArCS-2, a total of 294 patients with ACS and hyperglycemia were randomized to either intensive glucose control or conventional management (42). The target glucose levels were 85–110 mg/dL and <288 mg/dL, respectively. The primary endpoint was high-sensitive troponin *T*-value 72 h after admission. Glucose levels in the intensive arm were significantly lower than that of control arm within 36 h, but equalized by 72 h. Unexpectedly, there're no difference between the groups in the troponin *T*-values at 72 h. In contrast, a median follow-up of 5.1 years of the study reported higher rates of mortality at both 30 days and long term, suggesting intensive glucose control in the early phase of AMI resulted in persistent harmful effects (43). Compared to DIGAMI, BIOMArCS-2 had a more stringent target glucose level in the intervention arm. Although further analysis of BIOMArCS-2 didn't demonstrate an association between hypoglycemia and increased mortality, a lower glucose target might be responsible for the opposite results gained from DIGAMI and BIOMArCS-2.

Additionally, insights from the cardiovascular outcome trials of new glucose-lowering drugs, including Glucagon-Like Peptide 1 Receptor Agonists (GLP-1 RAs) and Sodium-Glucose Co-Transporter 2 (SGLT-2) inhibitors (44–46), indicated a new management strategy on hyperglycemia which focused on clinical outcomes directly instead of just glucose control itself. Despite protective effects of GLP-1 RAs and SGLT-2 inhibitors on ischemia heart proved in animal infarction models (47–51), few trials have been performed in humans in the ACS setting. A pilot study found that STEMI patients treated with exenatide at the time of PCI had improved salvage of myocardium (52). Similar findings were reported in ACS patients treated with liraglutide (53–55). However, patients enrolled in these studies were not required to be hyperglycemic. Empagliflozin, a SGLT-2 inhibitor, were reported to reduce LV mass and improve diastolic function in patients with ACS and diabetes (56). Nevertheless, further human studies are needed for evaluation of the cardiovascular outcome of both drugs in the presence of ACS with SIH.

So far, given limited results from clinical trials, there're no unified recommendations on the optimal glucose target and therapeutic strategy for SIH in the ACS setting. A scientific statement from AHA recommended initiation of intensive glucose control when plasma glucose was >180 mg/dL (6). In contrast, NICE recommendations suggested to manage hyperglycemia in ACS patients by keeping blood glucose levels below 198 mg/dL (57). The most recent ESC guidelines on management of non-STEMI/STEMI recommended it's reasonable to keep the blood glucose concentration <200 mg/dL (58, 59). Anyway, absolute avoiding of hypoglycemia is consistent across various statements and guidelines. As most ACS patients are hospitalized in intensive care units, intravenous insulin infusion with close blood glucose monitoring is the recommended glucose-lowering strategy.

## Mechanisms

Depending on baseline glucose metabolic status, the mechanisms underlying SIH could be very different (60). The development of SIH in patients without established diabetes mellitus in the context of ACS probably results from a combination of pancreatic β-cell dysfunction and acute insulin resistance (60, 61). Beta cell responsiveness was significantly related to ABG amongst patients with AMI (62). Furthermore, plasma proinsulin concentration and the proinsulin/insulin ratio were higher in AMI patients compared to control populations (63). These results indicated β-cell dysfunction might be prevalent among patients suffering AMI. Besides, glucose production is enhanced by upregulation of both gluconeogenesis and glycogenolysis. A complicated interplay of neurohormones and cytokines plays an important role in the development of hyperglycemia during ACS (64). In particular, excessive glucagon is the primary mediator of augmented glucogenesis. Sympathetic nervous system activation stimulates glucagon release, together with other anti-insulin hormones including cortisol and growth hormone, leading to hyperglycemia (65, 66). Cytokines, for example, tumor necrosis factor-α (TNFα), could promote gluconeogenesis *via* stimulation of glucagon production (67). Meanwhile, acute insulin resistance develops through two major pathways, including impaired post-receptor insulin signaling and downregulation of glucose transporter-4 (68). Both cytokines, such as TNFα and interleukin 1, and stimulation of β-adrenergic receptors can inhibit post-receptor insulin signaling (69–72). Overproduction of cortisol also reduces insulin-mediated glucose uptake (73). Additionally, insulin resistance promotes lipolysis because of a catabolic state. In turn, the resultant excessive circulating free fatty acids exacerbate insulin resistance by disrupting insulin signaling and glycogen synthase (74, 75).

**Figure 1:**
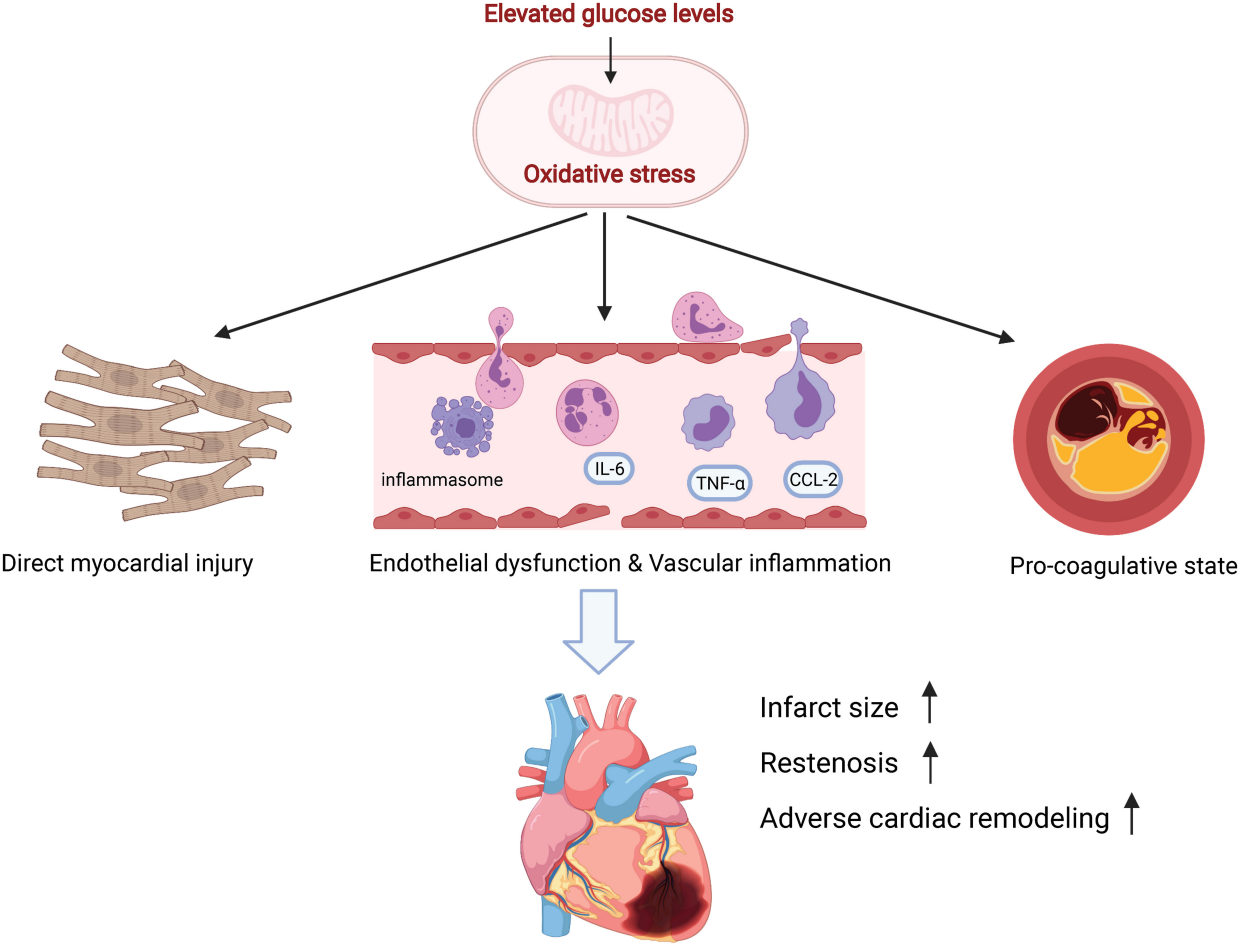
Postulated mechanisms underlying detrimental effects of SIH.

It's accepted that oxidative stress plays an important role in myocardial reperfusion injury as well as post-infarction remodeling (76, 77). Meanwhile, insights from both animal and human studies highlighted the role of increased oxidative stress in the pathophysiology of SIH (78–81). In turn, increased oxidative stress resulted in various tissue damaging *via* certain intracellular pathways, including the inflammatory and the non-oxidative glucose pathways (NOGPs) (60). Taking together, exacerbated oxidative stress during SIH might be a plausible mechanism responsible for additive subsequent detrimental effects in the ACS setting (Figure 1). First, acute hyperglycemia exerts a direct harmful effect on ischemic myocardium, probably *via* interfering with remote ischemic preconditioning (RIPerC). Kersten et al. showed that acute hyperglycemia abolished RIPerC induced cardioprotection and increased myocardial infarct size in a dose-dependent way (82). Similar finding was reported by Baranyai et al. in a rat model (83). However, some evidence suggested that chronic hyperglycemia reduced the infarct size and improved systolic function in rats after MI (84). Mechanisms underlying the cardioprotective effect of chronic hyperglycemia could be reduced cell necrosis, proinflammatory cytokines, and increased cell survival factors expression (84, 85). It seems that chronic hyperglycemia ahead of MI sets up a cellular preconditioning in response to acute rise of blood glucose. Secondly, both exacerbated vascular inflammation and endothelial cell dysfunction were implicated in the context of SIH (39, 86). Several studies showed an association of higher glucose levels with increased markers of vascular inflammation, including C-reactive protein, interleukin-6 and TNF-α (87, 88). Besides, hyperglycemia was reported to increase activation of prothrombotic factors, such as fibrinopeptide A and factor VII, and decrease plasma fibrinolytic activity (89–91). In an analysis of coronary thrombus from patients with STEMI, hyperglycemic patients showed a higher thrombus size, erythrocyte, fibrin, and macrophage levels (92). Finally, increasing studies implicated an association of SIH with post-infarct left ventricular systolic dysfunction (93, 94). Nevertheless, the underlying mechanisms need further illustration.

## Discussions

In this brief review, we discussed the definition, effects on clinical outcome, management, and pathophysiology of SIH in the context of ACS. A precise definition of SIH is helpful for designing interventional trials about glucose control in ACS patients. Only in this way, can we have high quality trials that shed lights on the nature of SIH. Therefore, we mainly focused on how to precisely define SIH. An optimal glucose metrics defining SIH should fulfill the following criteria that it correlates well with both short- and long-term outcomes regardless of the prior diabetic status. Unfortunately, a single glucose metrics seems unable to fulfill such criteria with present methodology. In the future, a combination of glucose metrics used to define SIH is reasonable and needs further investigations. We have fully understood that SIH is independently associated with adverse outcome of patients with ACS. However, it remains to be illustrated whether it's a marker of disease severity or a risk factor contributing directly to the poor clinical outcome. To address the issue, both clinical trials utilizing a unified precise definition of SIH and basic experiments revealing the underlying mechanisms are in demand. We suggest that researchers consider to set different glucose targets for patients with or without recognized diabetes mellitus in the future clinical trials targeting SIH in patients with ACS. With regards to underlying mechanisms, difference between the pathophysiological response of patients with or without previous persistent hyperglycemia should be taken into consideration.

## Author Contributions

ML, GC, and YF wrote the manuscript. XH revised the manuscript. All authors contributed to the article and approved the submitted version.

## Conflict of Interest

The authors declare that the research was conducted in the absence of any commercial or financial relationships that could be construed as a potential conflict of interest.
